# Treatment guidelines for rare, early-onset conditions associated with epileptic seizures: a literature review on Rett syndrome and tuberous sclerosis complex

**DOI:** 10.1186/s13023-023-02994-x

**Published:** 2024-02-26

**Authors:** S. Amin, B. Ruban-Fell, I. Newell, J. Evans, K. Vyas, C. Nortvedt, R. F. Chin

**Affiliations:** 1 Bristol Royal Hospital for Children, Research and Education Centre, Upper Maudlin St, Bristol, BS2 8AE UK; 2 Costello Medical, London, UK; 3 Costello Medical, Cambridge, UK; 4 GW Pharmaceuticals, London, UK; 5 Royal Hospital for Children and Young People, Edinburgh, UK; 6 Muir Maxwell Epilepsy Centre, Centre for Clinical Brain Sciences, Edinburgh, UK

**Keywords:** Seizures, Guidelines, Treatment, Rett syndrome, Tuberous sclerosis complex, Literature review, Rare diseases

## Abstract

**Background:**

Rett syndrome (RTT) and tuberous sclerosis complex (TSC) are two rare disorders presenting with a range of different epileptic seizures. Seizure management requires careful therapy selection, thereby necessitating development of high-quality treatment guidelines. This targeted literature review (TLR) aimed to characterise country-specific and international treatment guidelines available for pharmacological management of seizures in RTT and TSC.

**Methods:**

A TLR was performed between 25-Jan and 11-Mar 2021. Manual searches of online rare disease and guideline databases, and websites of national heath technology assessment bodies were conducted for the following countries: Australia, Canada, France, Germany, Israel, Italy, Japan, Spain, Switzerland, UK, and US as defined by pre-specified eligibility criteria. Search terms were developed for each condition and translated into local languages where appropriate. Eligible publications were defined as guidelines/guidance reporting pharmacological management of seizures in patients with RTT and TSC. Guideline development methodology, geographical focus, author information and treatment recommendations were extracted from guidelines. An author map was generated using R version 3.5.1 to visualise extent of collaboration between authors.

**Results:**

24 total guidelines were included, of which three and six contained only recommendations for RTT and TSC, respectively (some provided recommendations for ≥ 1 condition). Guideline development processes were poorly described (50% [12 guidelines] had unclear/absent literature review methodologies); reported methodologies were variable, including systematic literature reviews (SLRs)/TLRs and varying levels of expert consultation. Most (83% [20/24]) were country-specific, with guideline authors predominantly publishing in contained national groups; four guidelines were classified as ‘International,’ linking author groups in the US, UK, Italy and France. High levels of heterogeneity were observed in the availability of treatment recommendations across indications, with 13 and 67 recommendations found for RTT and TSC, respectively. For RTT, all treatment recommendations were positive and sodium valproate had the highest number of positive recommendations (Khwaja, Sahin (2011) Curr Opin Pediatr 23(6):633–9). All TSC treatments (21 medications) received either exclusively negative (National Organization for Rare Disorders (2019)) or positive (Chu-Shore et al. (2010) Epilepsia 51(7):1236–41) recommendations; vigabatrin received the highest number of positive recommendations (Kaur, Christodoulou (2019)).

**Conclusions:**

This review highlights the need for the development of international high-quality and comprehensive consensus-based guidance for the management of seizures with pharmacological therapy in RTT and TSC.

**Trial registration:**

Not applicable.

**Supplementary Information:**

The online version contains supplementary material available at 10.1186/s13023-023-02994-x.

## Background

Rett syndrome (RTT) and tuberous sclerosis complex (TSC) are rare, single-gene disorders that can present with autism, epilepsy and intellectual disability [1–3]. RTT is a progressive, developmental impairment disorder that almost exclusively affects females, with symptoms varying dramatically between patients [1]. TSC is a multi-system disorder characterised by formation of benign tumours in several organs and is associated with developmental delay and cognitive dysfunction [2]. Despite these distinct aetiologies, both conditions are known to cause epileptic treatment-resistant seizures from an early age [4], with a range of different seizure types observed in both cases [5, 6]. Many individuals with TSC initially manifest infantile spasms, although most other seizure types, including both focal and generalised, have also been associated with TSC [2]. Age of onset of epilepsy in RTT is typically later than TSC, with a mean age of 5 years [7], compared to < 2 years in TSC [8], with a cumulative risk of developing epilepsy of approximately 90% over the lifespan. The occurrence and remission of these seizures are highly heterogeneous [9], and it is very common for children with RTT to experience more than one seizure type, thereby complicating treatment decision-making [10]. In addition to epilepsy, severe breathing disturbances and non-epileptic events such as non-epileptic myoclonic jerks are also frequently present [11, 12].

The management of seizures in RTT and TSC is an important aspect of the overall management strategy of these conditions [7, 13–17]. Therapies must be chosen carefully to optimise seizure control, to reduce the risk of preventable injuries and complications associated with seizures and to improve patient quality of life (QoL) [18]. Early control of seizures is especially important in TSC as it is thought to prevent subsequent developmental epileptic encephalopathy and to reduce cognitive behavioural consequences [19, 20]. Pharmacological management with long-term anti-seizure medications (ASM) remains the primary seizure treatment for these disorders, but can be associated with significant side effects [2, 21–24]. Furthermore, there is a general lack of comparative studies examining the efficacy and safety of different ASMs used in monotherapy or combination treatment for these disorders [5, 25], and it is known that the efficacy of different ASMs in controlling seizures in RTT and TSC varies among individuals [5, 6, 26, 27]. Alongside medical treatments, non-pharmacological management of seizures, including ketogenic diets and vagus nerve stimulation may also be used in RTT [23], while in TSC, epilepsy surgery can be used [26]. Mammalian target of rapamycin (mTOR) inhibitors have also been highlighted as potential pharmacological treatments for TSC [27].

Given that seizures in RTT and TSC are often resistant to treatment (in that they are not adequately controlled despite the use of two or more appropriately chosen ASMs), treatment of these conditions often requires a combination of different medications [5, 6]. Treatment choice is therefore carefully considered and informed by a number of factors, including seizure type, the affected individual’s age, the severity of symptoms (as well as specific organ system involvement for TSC), presence or absence of learning disabilities in TSC and other comorbidities such as kidney angiomyolipomas [1, 2].

The selection of appropriate ASMs to manage the seizures attributed to these complex disorders is both challenging yet potentially highly beneficial for patients, and the development of appropriate treatment guidelines, in both national and international contexts, helps to provide clinicians with a clear and optimised management strategy that can be shared between specialists [28]. Moreover, these treatment recommendations often inform health technology assessment (HTA), regulatory body guidance, and payer coverage for treatments in some geographies, and thereby influence treatment licensing whilst affecting patients’ access to novel treatments [29, 30].

However, due to a lack of evidence within the literature, treatment guidelines for rare diseases are frequently difficult to find and of varying quality, [31, 32] despite their recognised contribution to improvements in quality of patient care [33]. Furthermore, as rare conditions are encountered infrequently by clinicians, rigorous treatment guidelines are particularly important to guide the management of seizures and co-morbidities in RTT and TSC [31]. Even if treatment guidelines are available, often they are not specific to the treatment of seizures within the rare condition in question, and may either provide guidance on the condition in general with a brief description of managing seizures, or conversely, may focus on seizure management in a wide range of conditions. A clear demand for robust, treatment guidelines has been highlighted in a user satisfaction survey undertaken by the Orphanet website (an online initiative which aims to provide high-quality information on rare diseases). In this survey, respondents reported interest in both having access to more clinical guidelines and review articles than were currently available and an expanded availability of resources from a wider geographical range [34].

Clinicians stand to benefit from the pooled expertise and evidence shared through author collaborations based on methods of robust evidence generation, such as systematic literature reviews (SLRs) and rigorous forms of expert consensus. However, national and international collaboration between guidelines developers is needed to develop high-quality recommendations and avoid duplication of effort [35, 36].

This targeted literature review (TLR) aimed to provide a multinational overview of available treatment guidelines for the pharmacological management of seizures in RTT and TSC, and their treatment recommendations. A TLR was chosen in order to search less standard sources than those typically seen in a systematic review in light of the fact that not all guidelines are published in traditional medical journals. More specifically, we aimed to:

1. Investigate the availability of region/country-specific and international treatment guidelines for RTT and TSC;
2. Describe guideline development methodologies;
3. Evaluate the extent of author collaboration through the development of an author network using included guidelines; and
4. Report the frequency of existing positive and negative treatment recommendations for RTT and TSC.

## Methods

### Search strategy

A TLR was performed between 25th January and 11th March 2021 to identify relevant treatment guidelines. The dates of searches and strategies used for each information source, which have been previously reported [35], were adapted for RTT and TSC and are summarised in Table S1, Additional File 1. Briefly, the following online information sources were manually searched in accordance with a pre-specified protocol: Google, Guideline Central, Orphanet, National Organisation for Rare Disorders (NORD), American Academy of Neurology (AAN), American Epilepsy Society (AES) and International League Against Epilepsy (ILAE). In addition, national HTA body websites for the following countries were also searched: Australia (Pharmaceutical Benefits Scheme), Canada (Canadian Agency for Drugs and Technologies in Health), France (Haute Autorité de Santé), Germany (Gemeinsamer Bundesausschuss), Israel (State of Israel – Ministry of Health), Italy (Agenzia Italiana del Farmaco [AIFA]), Japan (Ministry of Health, Labour and Welfare), Spain (Ministerio de Sanidad, Consumo y Bienestar Social), Switzerland (Bundesamt für Gesundheit), and United Kingdom (UK) (National Institute for Health and Care Excellence [NICE]. When searching the databases, search terms suitable for the database functionality were used, specific database features were accounted for, searches were filtered for guidelines where possible, and combinations of free-text and terms for each of the indications of interest were used as search terms.

### Review process

Criteria defined using a PICOS (Population, Intervention, Comparators, Outcomes, Study design) approach were used to screen identified records for eligibility and are presented in Table 1. These criteria were defined *a priori* in the review protocol, in order to reduce bias in the review and selection of records by reviewers. The review process has been previously reported [35]. In brief, guidelines or guidance publications were deemed eligible for inclusion if they reported on the pharmacological management of seizures in patients with RTT or TSC in the countries of interest.

**Table 1:** Eligibility criteria

Modified PICOS domain	Inclusion criteria	Exclusion criteria
Population	Patients with the following epileptic conditions: • Rett syndrome • Tuberous sclerosis complex	Conditions other than those listed
Intervention	Any	None
Outcomes	The document must have discussed the management of the conditions of interest in terms of pharmacological treatment pathways for routine seizure control	• Documents that did not discuss the management in terms of pharmacological treatment pathways • Emergency medication and surgical guidelines
Publication type	Guidelines or guidance documents	Publications other than guidelines
Other considerations	Specifically produced for use in: • EU5 countries (UK, Germany, Spain, Italy, France) • Japan • Australia • Switzerland • Israel • US • Canada International guidelines (i.e. guidelines produced for multiple countries that included or potentially included the countries of interest, or guidelines that did not specify which countries they pertained to)	Produced specifically for use in countries that were not of interest

PICOS, Population, Intervention, Comparators, Outcomes, Study design; UK, United Kingdom; US, United States

Publications which were informed by rigorous methods, had multiple authors or explicitly indicated that certain treatments were ‘recommended’ were defined as guidelines. The review also captured technology appraisal guidance developed following HTAs, as well as guidelines produced by HTA bodies. The review was conducted in accordance with a pre-specified protocol in order to ensure a comprehensive review of all relevant sources and to reduce sources of bias in screening and extraction. Search results were screened, using the pre-specified protocol, by a single reviewer, except where applicability of the search criteria was unclear, in which case the record was assessed by a second reviewer.

### Data extraction and analyses

A pre-defined extraction grid was used to extract relevant data from the guidelines that were included in this review, as described previously [35]. In brief, the following information was extracted: author names and affiliations; publication date and planned revision date; the methodology of guideline development; population(s) addressed; pharmacological treatment recommendations; references to other treatment recommendations. Eligible publications were classified as “International” if they were developed either for multiple countries or did not specify to which countries they pertained. If neither of these criteria were fulfilled, regional author affiliations were used to determine nationality of the guideline.

The country-specificity of identified guidelines, methodology of guideline development, references made to treatment recommendations in other sources and the cross-referencing of treatment recommendations made within other guidelines were analysed descriptively in Microsoft Excel^®^.

The extent of collaboration between authors who had authored > 1 guideline both at a national and international level was measured by mapping and visualising the authors involved in the development of each guideline into a network. This visualisation was developed using the programming language R version 3.5.1.

An individual ASM that was recommended for use in a specific indication, irrespective of the line of treatment or whether the treatment was adjunctive was defined as a positive recommendation. An individual ASM treatment that was highlighted as a potential option by a guideline but whose use was recommended against (for any reason) in a specific indication, irrespective of the line of treatment or whether the treatment was adjunctive, was considered a negative recommendation.

## Results

### Characteristics of included guidelines

**Fig. 1:**
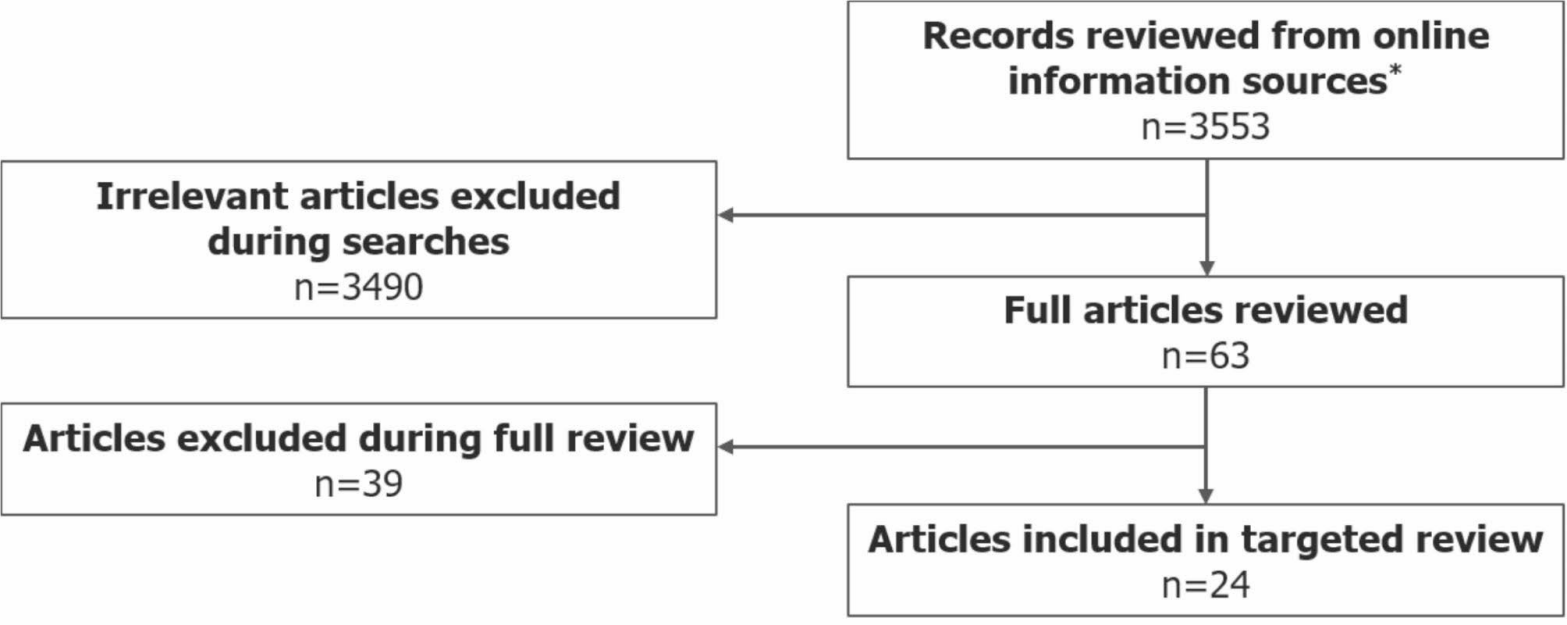
Literature review flowchart. *Online information sources included: Guideline Central, National Organization for Rare Disorders (NORD), American Academy of Neurology (AAN), American Epilepsy Society (AES), International League Against Epilepsy (ILAE), Orphanet, Google, National Institute for Health and Care Excellence (NICE), Pharmaceutical Benefits Scheme (PBS), Canadian Agency for Drugs and Technologies in Health (CADTH), Ministerio de Sanidad, Consumo y Bienestar Social (MSCBS), Agenzia Italiana del Farmaco (AIFA), Haute Autorité de Santé (HAS), Gemeinsamer Bundesausschuss (G-BA), Bundesamt für Gesundheit (BAG), State of Israel – Ministry of Health, Ministry of Health, Labour and Welfare (MHLW)

**Fig. 2:**
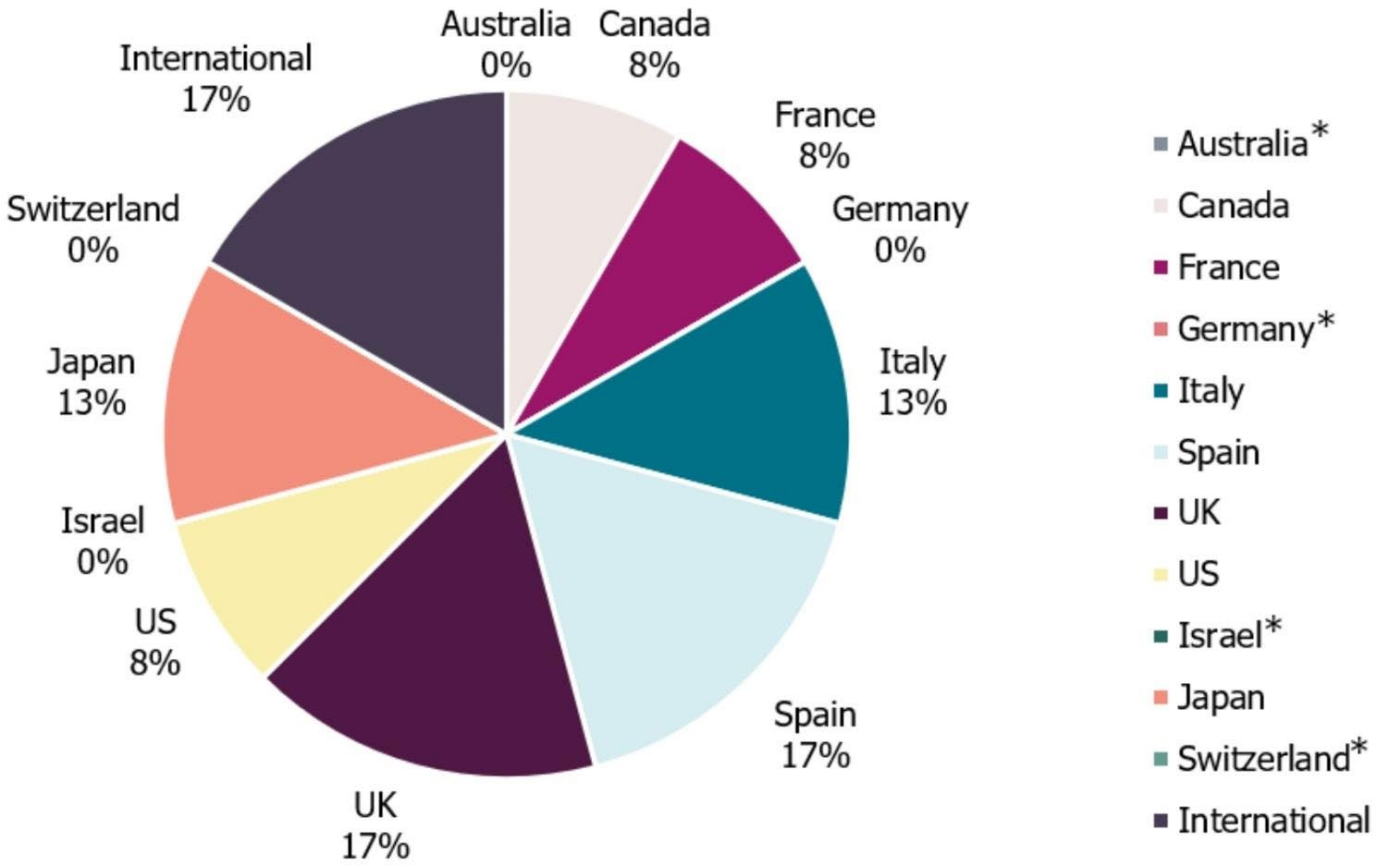
Geographies of identified guidelines. *No guidelines were identified for use in Israel, Switzerland, Germany or Australia. The geography of guideline use refers to the country for which the guidance was specifically developed. UK, United Kingdom; US, United States

A total of 24 eligible records with publication dates ranging between November 2005 and January 2021 were identified following removal of duplicate results (Fig. 1; Table S2, Additional File 1). Most guidelines were country-specific (i.e. recommendations were intended for patients in a specific country), with only four guidelines (17%) considered to be “International” (Fig. 2). Guidelines were identified in 7/11 (64%) of regions that were pre-defined in the review criteria (‘International’ guidance was included as one region in this case). Otherwise, the individual countries with the highest number of identified guidelines were the UK (17% [4/24]), Spain (17% [4/24]), Italy (13% [3/24]) and Japan (13% [3/24]). No guidelines were identified for use in Israel, Switzerland, Germany or Australia. Of the guidelines identified, five were specifically developed for regions within one of the countries of interest (21% [5/24]): Two Canadian guidelines were developed specifically for the provinces of British Columbia [37] and Ontario [38], one UK guideline was created for use in Scotland [39], one Italian guideline was developed for the region of Tuscany [40] and one of the four Spanish guidelines identified was created specifically for the region of Andalusia [41]. None of the US guidelines identified (8% [2/24]) were for use at the state level.

### Evidence base and methodology for guideline development

**Fig. 3:**
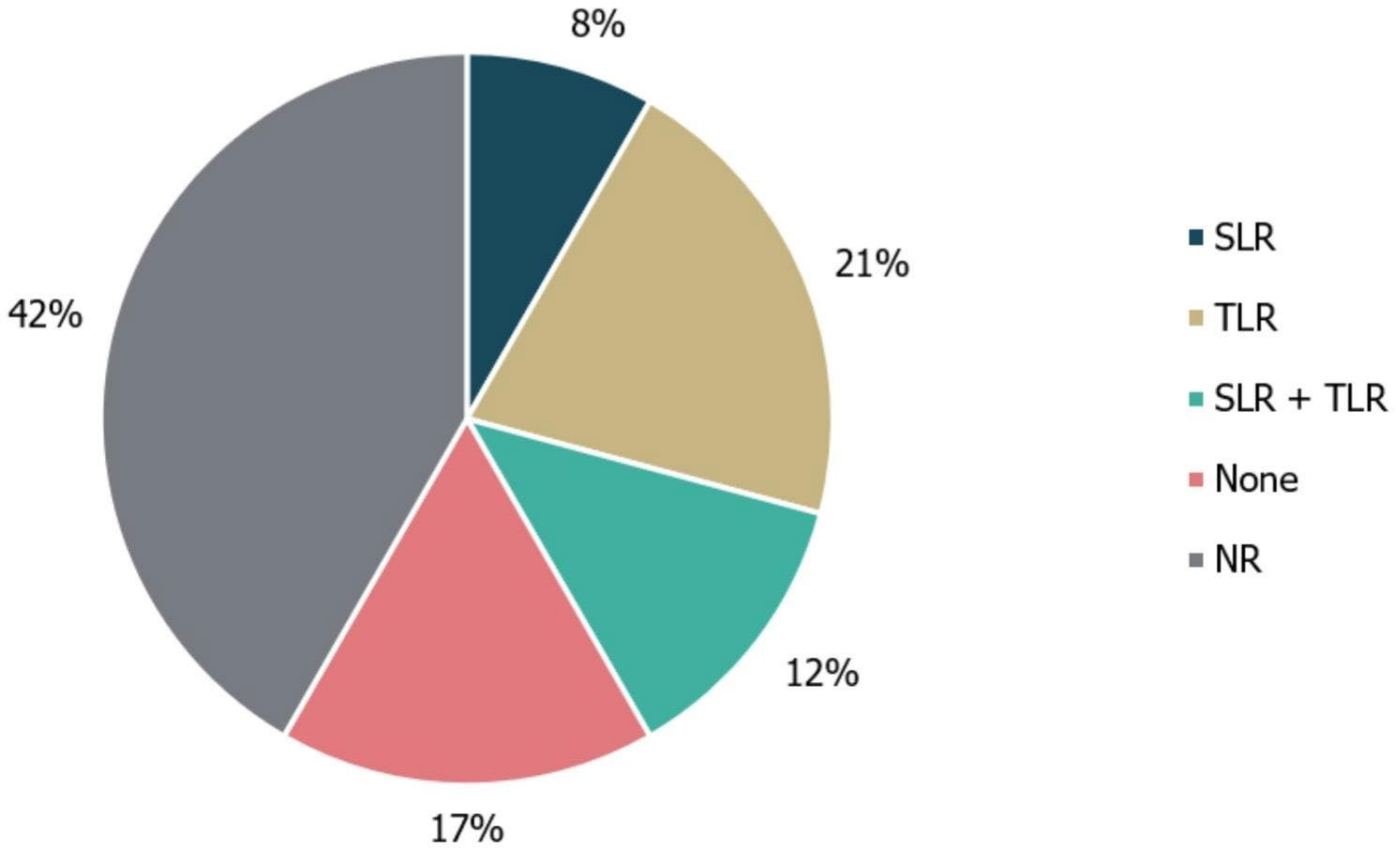
Types of literature review performed to inform guideline development. ‘None’ refers to guidelines in which a literature review was explicitly not used. NR, not reported; SLR, systematic literature review; TLR, targeted literature review

**Fig. 4:**
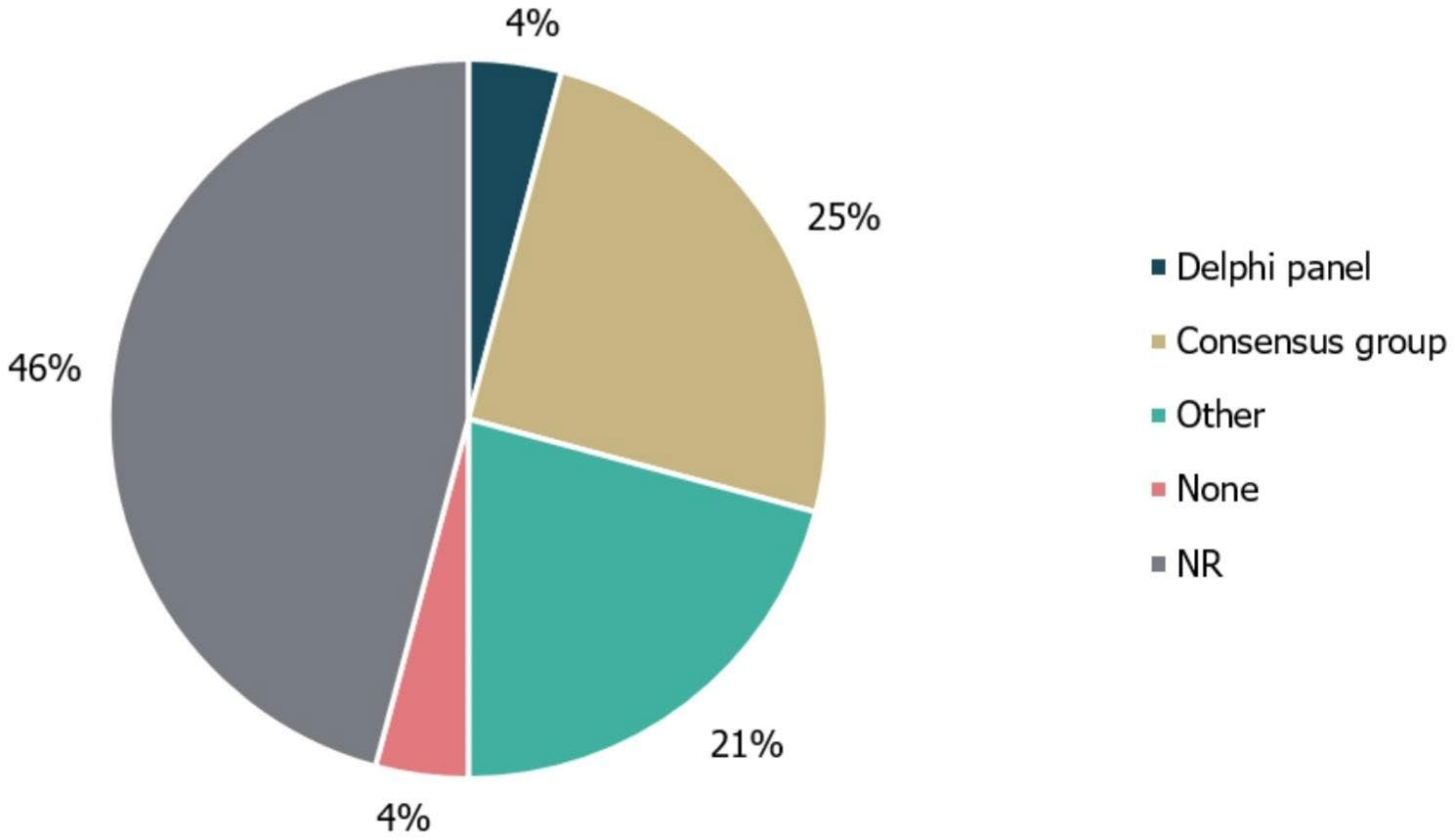
Types of expert consultation performed to inform guideline development. ‘None’ refers to guidelines in which expert consultation was explicitly not used; ‘Other’ refers to working groups or targeted expert interviews. NR, not reported

Among the 24 identified guidelines, 10/24 (42%) did not specify whether guideline development was informed using literature reviews. Notably, none of the guidelines for RTT detailed a guideline development methodology. Additionally, 4/24 guidelines (17%) explicitly did not use a literature review to develop their recommendations. Of the remaining guidance documents, 7/21 involved literature searches (2/24 [8%] SLR; 5/24 [21%] TLR); of these, 3/24 (12%) used a combination of both methods (Fig. 3). No details regarding use of expert consultation to develop recommendations were reported by 11/24 guidelines (46%). A Delphi panel (a structured and iterative survey technique used to gather consensus on specific issues from a group of experts) was used to inform the development of 1/24 (4%) guidelines, while 6/24 (25%) guidelines were based on formal consensus group exercises. Other forms of expert consultation, including working groups or targeted expert interviews, were used to develop the remaining five guidelines (21%; Fig. 4). While the use of a combined development approach consisting of a literature review and expert consultation was reported by 9/24 (38%) of guidelines, none explicitly utilised the combination of an SLR and a Delphi panel.

**Fig. 5:**
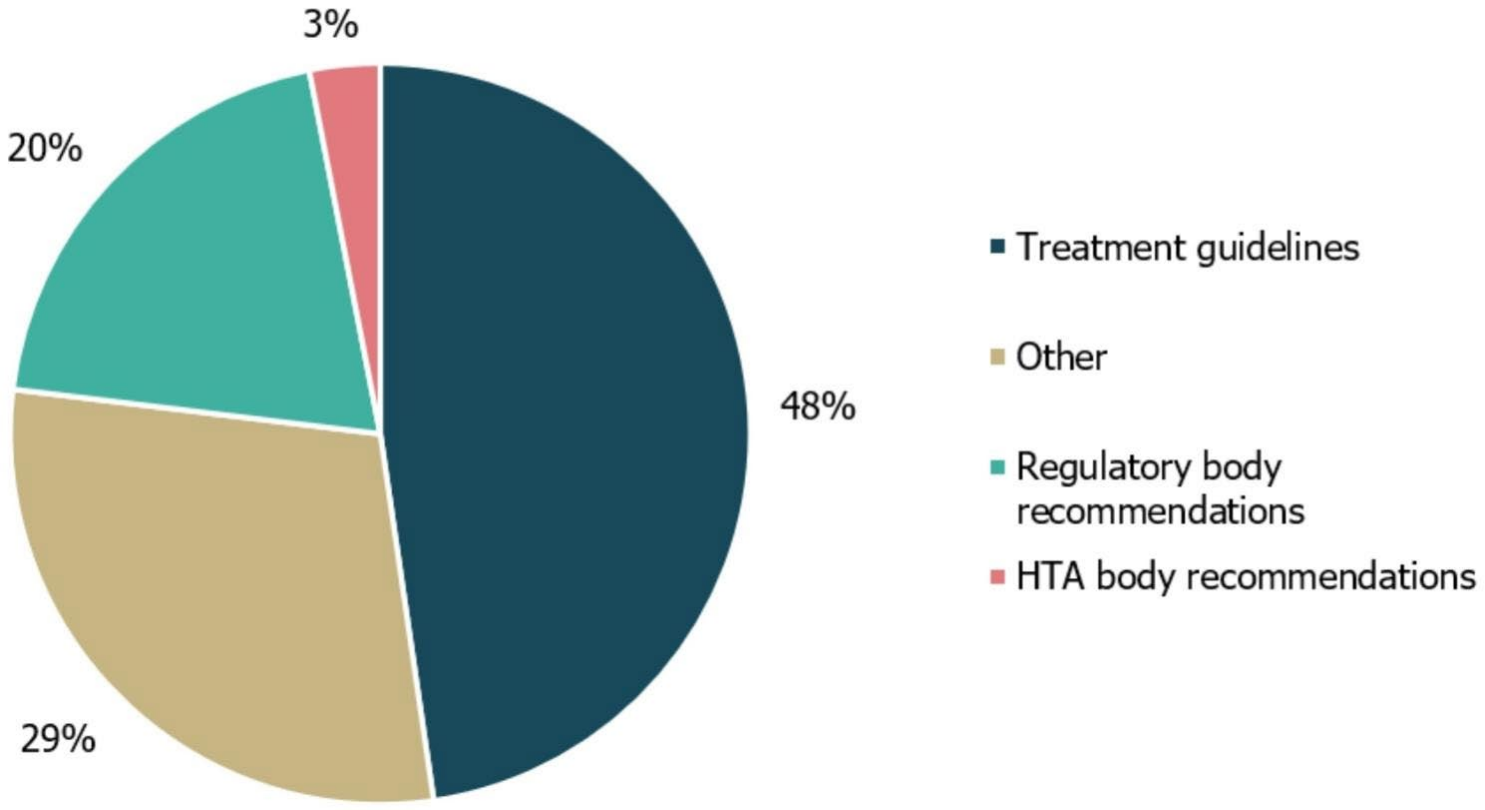
Guideline cross-referencing to other treatment guidelines and regulatory/HTA recommendations. Cross-referencing refers to the number of different treatment guidelines, regulatory body recommendations, HTA body recommendations or other references that were cited within the guidelines identified in this study, either in the body of the guideline text or in accompanying reference lists. ’Other’ references included a Cochrane systematic literature review, and informational websites about RTT or TSC. HTA, health technology assessment; RTT, Rett syndrome; TSC, Tuberous sclerosis complex

The included guidelines mainly cited other treatment guidelines for TSC and RTT (31/65 citations; 48%) or other compiled literature sources such as literature reviews (19/65; 29%). The referenced literature reviews largely consisted of SLRs included in the Cochrane Database of Systematic Reviews (13/19; Fig. 5). Regulatory body recommendations comprised 13/65 of the citations; of these, two were made to HTA body recommendations. The UK’s NICE guidance on the diagnosis and management of epilepsies (CG137) [42], an SLR from the Cochrane Database of Systematic Reviews on the treatment of infantile spasms [43] and an SLR from the Cochrane Database of Systematic Reviews on the treatment of Lennox-Gastaut syndrome were the most frequently referenced documents (five, five and six times, respectively) [44].

### Extent of author collaboration

**Fig. 6:**
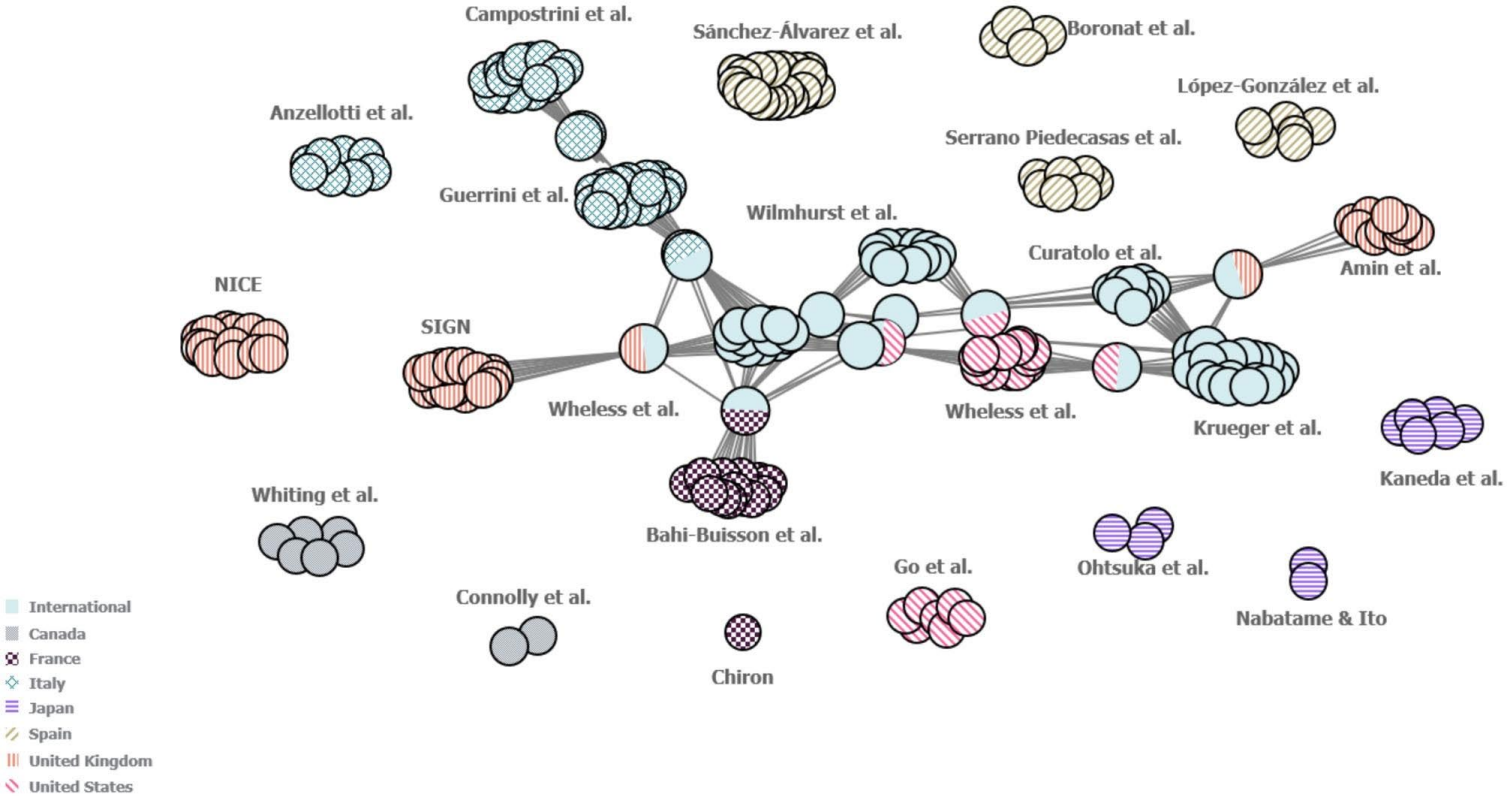
Map of collaboration between the author groups of included guidelines. Each individual circle represents one author of a guideline. Each ‘cluster’ represents the group of authors that developed one guideline. Each cluster is labelled with the names of its respective first author(s). Guidelines which share one or more authors between them are connected by grey lines, with single circles between guideline clusters representing the individuals who authored both guidelines in question. Guidelines were classified as “International” if they were developed either for multiple countries or did not specify to which countries they pertained. Guidelines for which author names were not reported have not been included in this figure. NICE, National Institute for Health and Clinical Excellence; SIGN, Scottish Intercollegiate Guidelines Network

A visualisation of the network of authors involved in developing each of the guidelines identified in this study (including those developed for both RTT and TSC) was developed to determine the extent of national and international collaboration. Using this author map, connections were identified between authors involved in the development of international treatment guidelines and US, UK, French and Italian guideline author groups (Fig. 6). Other regional guidelines were developed within contained national groups.

### Treatment recommendations for Rett syndrome

**Fig. 7:**
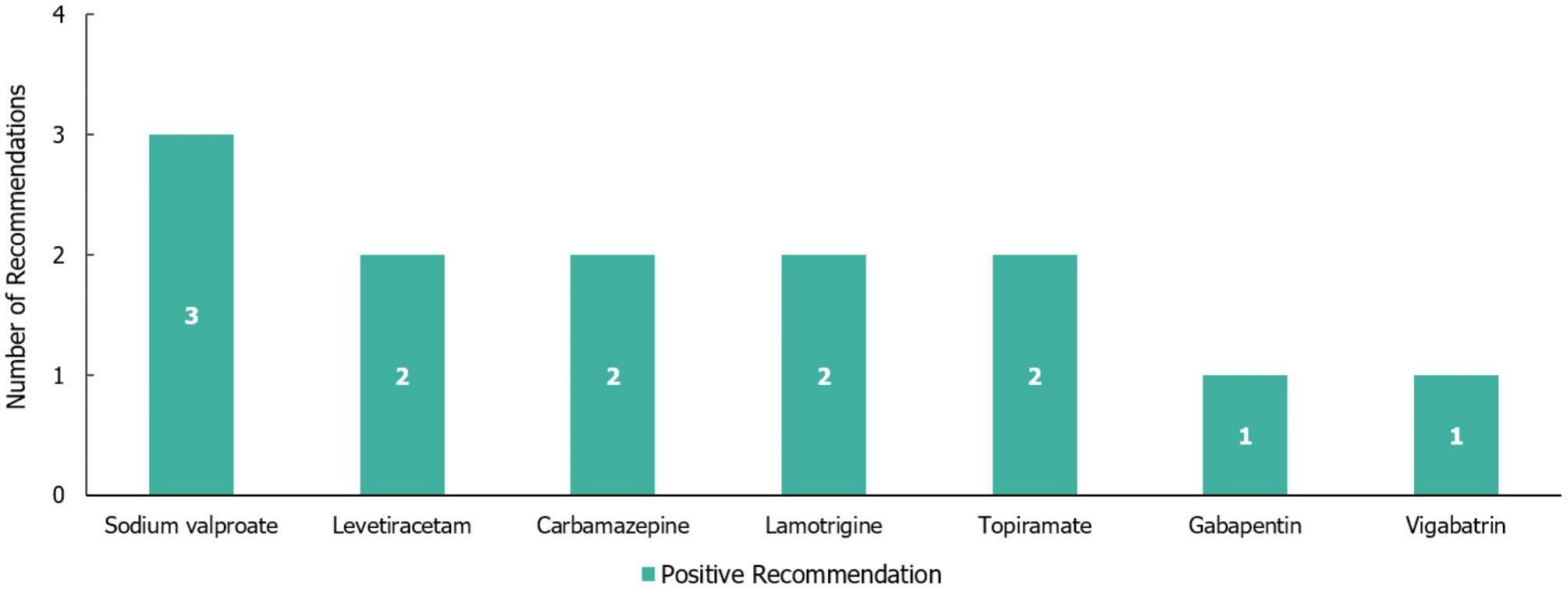
Treatment recommendations for Rett syndrome. N = 13 (13 positive and 0 negative recommendations) from three guidelines. Positive recommendation: use of an individual ASM treatment that was recommended for use in a specific indication, irrespective of the line of treatment (e.g. first line) or whether the treatment was adjunctive; Negative recommendation: an individual ASM treatment that was highlighted as a potential option by a guideline but not recommended by a guideline for use (for any reason) in a specific indication, irrespective of the line of treatment or whether the treatment was adjunctive

Only three guidelines were identified that made recommendations for the symptomatic treatment of seizures in RTT, and among these, a total of 13 individual treatment recommendations were made (irrespective of the line of treatment; see Fig. 7), all of which were positive. Overall, seven different medications were included in treatment recommendations for RTT, of which sodium valproate had the highest number of positive recommendations (3; Fig. 7). No recommendations for a specific line of treatment were made for use in RTT; however, two recommendations for specific seizure types were made, both of which were positive recommendations for the treatment of myoclonic seizures (one for levetiracetam and one for topiramate).

### Treatment recommendations for tuberous sclerosis complex

**Fig. 8:**
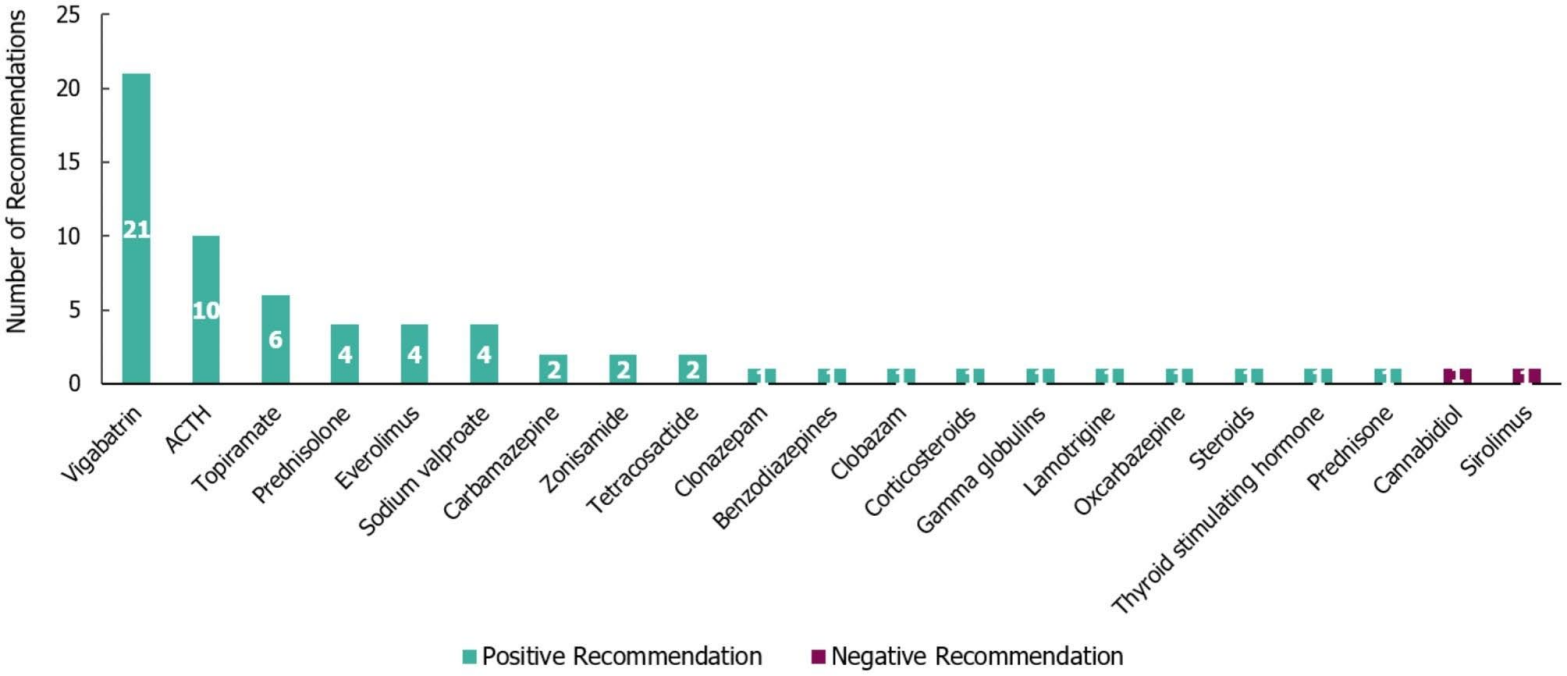
Treatment recommendations for tuberous sclerosis complex. N=67 (65 positive and two negative treatment recommendations) from 22 guidelines. Positive recommendation: use of an individual ASM treatment that was recommended for use in a specific indication, irrespective of the line of treatment (e.g. first line) or whether the treatment was adjunctive; Negative recommendation: an individual ASM treatment that was highlighted as a potential option by a guideline but not recommended by a guideline for use (for any reason) in a specific indication, irrespective of the line of treatment or whether the treatment was adjunctive. ACTH, adrenocorticotropic hormone

A total of 67 individual treatment recommendations, irrespective of the line of treatment, were made within the 22 guidelines identified for TSC (Fig. 8). The vast majority of treatment recommendations for TSC were positive (65/67 [97%]). Vigabatrin had the highest number of positive recommendations (21/65 [32%]) and was recommended for infantile spasms. The other medications which received a high number of positive recommendations were adrenocorticotropic hormone (ACTH; 10), topiramate [6], prednisolone [4] and sodium valproate [4]. Cannabidiol and sirolimus both received one negative recommendation each based on a lack of current evidence regarding their use.

The vast majority (65/67) of treatment recommendations for TSC were positive. Of these, 65% (42/65) were treatment line-specific (23 for first-line, 19 for second-line; Table S3, Additional File 1). The highest number of positive recommendations specific to first-line treatment was received by vigabatrin [15], while ACTH received the highest number of positive recommendations specific to second-line treatments [6]. There were no negative recommendations specific to treatment lines made for TSC.

Additionally, 90% of the total treatment recommendations (60/67) for TSC were specific to seizure type. Of these, 59 were positive recommendations and the majority (46; including 17 recommendations for vigabatrin and 10 for ACTH) were related to infantile spasms. Positive seizure type-specific recommendations were also made for focal seizures [9] and treatment-refractory [2] seizure types. One negative seizure type-specific recommendation was made for sirolimus in relation to treatment-refractory TSC.

## Discussion

This review provides a geographically diverse overview of available guidelines and their treatment recommendations for seizure management in RTT and TSC, with guidance documents from eleven countries across Europe, North America and Asia Pacific. In summary, the review identified a paucity of international guidelines; a limited use of ‘gold standard’ methodologies used in guideline development; a lack of international collaboration between author groups; and a low number of overall treatment recommendations for RTT.

Interestingly, the majority of guidelines were country-specific (four guidelines were classified as ‘International’). In addition, a reasonable proportion of the guidelines were specifically developed for a particular region within a given country, which may reflect differing geographical medication availabilities. However, despite this finding, there was no major divergence in recommendations between countries, suggesting a general consensus amongst guideline developers across the globe.

SLRs are considered the ‘gold standard’ for evidence synthesis, and the Delphi methodology is recommended for use in healthcare settings as a reliable means of determining consensus for defined clinical problems [45–47]. Despite this, this review identified a lack of guideline development approaches combining these two approaches in addition to a wide variety of methodologies used in general. Fewer than 50% of the guidelines specified that their development process was informed by literature reviews (42% [10/24]), and only half of the guidelines were developed through expert consultation (50% [12/24]), which highlights a need for further clarity and standardisation in the reporting and development of additional guidance using rigorous methodological processes. In addition, available tools to improve guideline reporting, such as the AGREE checklist [48], could be utilised more frequently to align and improve guideline development practice for these rare diseases [32].

Notably, detail on the methodology of guideline development was entirely absent for the three RTT guidelines, which is of particular concern given the limited literature available for treating this rare disease [49]. Conversely, most TSC guidelines were developed using a SLR or TLR, in combination with ‘other’ forms of expert consultation (such as working groups or targeted expert interviews), as opposed to the preferred combination of an SLR and Delphi panel. This may reflect the relatively large time and resource burden associated with these study types, problems which are likely to be more pronounced in the context of rare diseases [50].

Links between author groups who published the four International guidelines (Curatolo et al., Krueger and Northrup, Wheless et al. and Wilmshurst et al.) [24, 51–53] and author groups who published guidelines from the US, UK, Italy and France were identified. This suggests a reasonably well-defined network for TSC between the US and Europe, whilst highlighting the need for outreach and inclusion of author groups outside these regions, as international guidelines written solely by authors in high-income countries are unlikely to be suitable for most clinicians and patients around the world. Further, only one guideline (Bahi-Buisson et al.) [54] within this author collaboration network was for RTT. Despite the general consensus observed among the included guidelines, additional communication between national expert groups by pooling clinical expertise, e.g. via supra-national bodies, could potentially help to address the current lack of international guidelines for these disorders, particularly for RTT [55]. Similarly, further international and national guidance could benefit regions where no national guidelines are available to help inform the development of local guidelines.

This review highlighted the relatively limited number of treatment guidelines for RTT and TSC, which may reflect the critical need for treatments and the difficulty of developing treatment guidelines for rare diseases in general, due to low disease prevalence and patient population heterogeneity [56–58]. Consequently, there is an urgent need to develop additional up-to-date treatment guidelines for both RTT and TSC. The particularly low number of available treatment guidelines for RTT may reflect the difficulty in identifying the most appropriate ASM treatment according to seizure type and a patient’s individual needs [5]. This issue is compounded by a lack of comparative studies and the large number of different ASMs that must be evaluated, which is highlighted by the conflicting results found in previously conducted retrospective analyses.

For TSC, the most positively recommended treatment in the review reported here (vigabatrin) aligned with the ASM treatment recommendations made by the NICE guidance CG137 for infantile spasms [59]. However, there was no specific guidance in this document for the use of ASMs in RTT [59]. Similarly, the low number of available treatment guidelines for TSC may reflect the complexity of seizure management in this disorder, given that the efficacy of different ASMs can vary in different individuals and subsequently a combination of different medications is often required [2]. However, patients with TSC may have access to alternative treatment options to control seizures, such as surgery and vagal nerve stimulation [2]. Notably, there was a lack of negative recommendations for both of these disorders; only two such recommendations were identified (one each for the use of cannabidiol and sirolimus in TSC), which were due to lack of current evidence rather than a lack of efficacy.

The lack of recommendations for specific lines of treatment for RTT suggests a need to provide further clarity on the most suitable ASM regimen for patients with this disorder, and further guidance on which treatments should be prioritised or preferred over others. This finding may be due to a paucity of randomised controlled trials, the high cost of developing rare disease medicines, and the ethical challenges of conducting such studies on the developing brain [60]. The five most frequently recommended ASMs for RTT in this review are consistent with findings from other studies [7, 61–63]. However, as only a small number of patients have been included in reports addressing the effectiveness of newer ASMs, such as lamotrigine, levetiracetam, and topiramate (combined with the fact that new ASMs are likely to be developed) the number of available treatment recommendations for these medications may increase as further research is conducted [5].

Other key areas in which guidance was not identified included management of Sudden Unexpected Death in Epilepsy (SUDEP). Children with RTT in particular are at higher risk of sudden death, due to developmental delay, generalised seizures and use of polypharmacy in treating the condition [64]. Despite this, we did not find any discussion or guidance around SUDEP in this review, which suggests that further guidance is needed on this topic.

The high proportion of treatment recommendations for TSC that were seizure type-specific (88% [59/67]) may reflect the varied seizure types that can be experienced by patients with this disorder, such as infantile spasms, which often require different treatment approaches [65]. In contrast, only 15% (2/13) of recommendations for RTT were seizure type-specific (these were both myoclonic), despite the fact that generalised tonic-clonic and complex partial seizures are considered to be the most common seizure type in patients with RTT.

Notably, vigabatrin received exclusively positive treatment recommendations in TSC, and a comparatively large proportion of these were treatment line- and seizure type-specific. However, the consensus identified around positive recommendation for vigabatrin in TSC, despite known safety concerns (such as drowsiness, fatigue, nausea, behaviour, mood changes, and visual field defects in some adults), highlights an unmet need with regard to patient QoL [66].

Despite the approval of the mTOR inhibitor, everolimus, as adjunctive therapy for TSC-associated partial onset seizures, this medication received a low number of recommendations in this review [67]. This is most likely due to the fact that guidelines can take time to develop following marketing authorisation of a medication. However, additional positive treatment recommendations may be expected for everolimus managing seizures, as a phase III, randomised, double-blind, placebo-controlled study in 2016 has shown that adjunctive treatment significantly reduces seizure frequency with a tolerable safety profile compared with placebo in patients with TSC and treatment-resistant seizures [68]. Indeed, the 2021 guidelines from the International TSC Consensus Group, which were recently updated from the 2013 guidelines included in this review, recommend both everolimus and cannabidiol as adjunctive therapies for non-infantile seizures in TSC. Moreover, these recommendations are based on Category I (e.g. defined as highest quality) evidence [69].

In addition, many of the treatments that were widely recommended in this review have no specific licence for RTT or TSC. For example, the ASMs that received positive recommendations for RTT are either licensed for treating all forms of epilepsy (e.g. sodium valproate [70]) or for specific seizure types (e.g. carbamazepine, [71] lamotrigine [72], topiramate [73], and levetiracetam [74]), rather than specifically for treating seizures in this disorder. For sodium valproate specifically, safety concerns have been published by NICE and the AIFA since development of the guidelines identified in this review [75, 76], (specifically for girls and young women of childbearing age around the risk of polycystic ovarian syndrome), suggesting clinicians are moving away from use of this treatment. In TSC, apart from everolimus and an oral solution of cannabidiol, which have been approved by the Food and Drug Administration (FDA) [77, 78] and the European Medicines Agency (EMA) [79, 80] as adjunctive treatments for seizures, other ASMs that received a high number of positive recommendations for TSC are licensed more generally for specific seizure types, but not for this specific indication (e.g. vigabatrin, [66] topiramate [73], and carbamazepine [71]).

Some limitations were identified as a result of the targeted nature of the review. A single reviewer assessed the eligibility of all records; when the applicability of the inclusion criteria was unclear, a second independent reviewer adjudicated the decision. Use of an additional reviewer when assessing eligibility may have helped to reduce bias and inaccurate application of inclusion/exclusion criteria [81]. Additionally, this literature review searched sources not typically used in a systematic review, including those found using Google, medical society and guideline developer websites. The decision to focus on these sources minimised the risk of missing indexing local guidelines, given that not all guidelines are published in traditional medical journals or in the English language. Despite these sources being less standard for a literature review, they returned a large number of specific records and provided a multinational overview of the available guidelines and their treatment recommendations in the absence of previously conducted analyses. This review further aimed to report on guidance documents from a broad sample of countries likely to be influential in the development of treatment guidelines. Due to focus of this review on Australia, Canada, France, Germany, Israel, Italy, Japan, Spain, Switzerland, the UK and the US only, the results may not fully represent the international landscape of treatment guidelines for RTT and TSC.

Another limitation of the review was that additional detail on ASM efficacy by patient age group was not captured, which previous research has shown to be relevant in the treatment of RTT; sodium valproate and carbamazepine were effective in patients who presented seizures within the typical age range (4–5 years), while lamotrigine (LTG) was effective for patients in whom epilepsy started later [16]. In addition, the time period of the review meant that treatment guidelines published after March 2021 were not captured. For example, further recommendations in Rett Syndrome have been published, such as the Rett Syndrome Health Checklist, published in 2021, by the patient advocacy and research organisation Reverse Rett [82]. The treatment recommendations identified in this review should therefore be interpreted in the context and date that they were made (all identified papers were published between December 2005 – January 2021), as new research and medication approvals may necessitate updates to treatment guidelines. Additional reviews could also be conducted to investigate the wider management of these disorders, including emergency treatment of seizures and surgical interventions for TSC.

## Conclusion

This review highlights the need for the development of further international, high-quality and comprehensive consensus-based guidance, influenced by a more diverse range of geographical regions, for the management of seizures with pharmacological therapy in RTT and TSC. In addition, the lack of treatment line-specific and seizure type-specific treatment recommendations for RTT highlights an urgent need for further guidance on selecting an appropriate ASM regimen for this disorder to optimise the management of seizures.

### Electronic supplementary material

Below is the link to the electronic supplementary material.

**Additional file 1:** Supplementary tables

## Data Availability

The data supporting the conclusions of this article are included within the article and its additional files.
