# Supplementary material for: Treatment guidelines for rare, early-onset conditions associated with epileptic seizures: a literature review on Rett syndrome and tuberous sclerosis complex
Source: Orphanet J Rare Dis. 2024 Feb 26;19:89. doi: 10.1186/s13023-023-02994-x (PMC10895812; doi:10.1186/s13023-023-02994-x)
Supplement: Supplementary file 1 — Additional file 1: Supplementary tables [file 13023_2023_2994_MOESM1_ESM.docx]

**SUPPLEMENTARY TABLES**

Table S1. Information sources and search strategies

| Information source | URL | Search date(s) | Search strategy^a^ |
| --- | --- | --- | --- |
| Guideline Central | [https://www.guidelinecentral.com](https://www.guidelinecentral.com/) | 28^th^ January 2021 | The webpage was searched for the following terms (using the general search function):  *“tuberous sclerosis complex”, “bourneville”, “rett”* |
| National Organization for Rare Disorders (NORD) | [https://rarediseases.org](https://rarediseases.org/) | 28^th^–29^th^ January 2021 | The webpage was searched for the following terms (using the advanced search function, searching ‘Rare Disease Database’ and ‘Physician Guides’ categories):  *"tuberous sclerosis complex”, “bourneville”, “rett”* |
| International League Against Epilepsy (ILAE) | <https://www.ilae.org> | 28^th^ January 2021 | The webpage was searched for the following terms (using the general search function):  *“tuberous sclerosis complex”, “bourneville”, “rett”* |
| Orphanet | [https://www.orpha.net](https://www.orpha.net/consor/cgi-bin/Disease_Search.php?lng=EN) | 28^th^ January 2021 | The webpage was searched for the following terms (using the “Disease name” setting):  *"tuberous sclerosis complex”, “bourneville”, “rett”* |
| American Academy of Neurology (AAN) | <https://www.aan.com> | 25^th^ January 2021 | The webpage was searched for the following terms (using the general search function):  *“tuberous sclerosis complex”, “bourneville”, “rett”* |
| American Epilepsy Society (AES) | <https://www.aesnet.org> | 1^st^ February 2021 | The webpage was searched for the following terms (using the general search function):  *“dravet", “SMEI”, “severe myoclonus epilepsy of infancy”, “severe myoclonic epilepsy of infancy”, “lennox gastaut”, “cdkl5 deficiency”, “CDD”* |
| Google | [www.google.com](http://www.google.com) | 10^th^–17^th^ February 2021 | *"tuberous sclerosis complex”, “bourneville”, or “rett”* AND *“guideline” or “guidance”* AND *“United Kingdom”, “UK”, “Australia”, “Canada,” “US”, “United States”, “America”, “American” or “international”* |
|  | [www.google.es](http://www.google.es) | 11^th^–15^th^ February 2021 | *"esclerosis tuberosa”, “enfermedad trastorno bourneville”, “rett”* AND *“epilepsia directrices guia” or “directrices guia”* AND *“espana”* |
|  | [www.google.it](http://www.google.it) | 15^th^ February 2021 | *"Sclerosi tuberosa”, “Sindrome di Bourneville”, “Sindrome de Rett”* AND *“guida” or “Linee guida”* AND *“Italia”* |
|  | [www.google.fr](http://www.google.fr) | 18^th^ February 2021 | *"La sclérose tubéreuse de Bourneville”, “STB”, “Syndrome de Rett”* AND *“conseil” or “lignes directrice”* AND *“France”* |
|  | [www.google.de](http://www.google.de) | 16^th^ February 2021 | *"Tuberöse Sklerose”, “Bourneville”, “rett”* AND *“richtlinie” or “leitlinie”* AND *“Deutschland”* |
|  | [www.google.ch](http://www.google.ch) | 16^th^ February 2021 | *"Tuberöse Sklerose”, “Bourneville”, “rett”* AND *“richtlinie” or “leitlinie”* AND *“Schweiz”* |
|  | [www.google.il](http://www.google.il) | 11^th^–15^th^ February 2021 | *"tuberous sclerosis complex”, “bourneville”, “rett”* AND “*guideline” or “guidance*” AND “*Israel*” |
|  |  |  | תסמונת רט  טוברוס סקלרוזיס  AND  קו מנחה  הדרכה  AND  ישראל |
|  | [www.google.jp](http://www.google.jp) | 16^th^ February 2021 | (レット症候群), (Rett症候群), AND (ガイドライン, ガイド), (指導), or (指針) AND (アドバイス) |
| National HTA Bodies/Medicine Agencies for Countries of Interest | | | |
| National Institute for Health and Care Excellence (NICE) | [https://www.nice.org.uk](https://www.nice.org.uk/) | 25^th^ January 2021 | The webpage was searched for the following terms (using the general search function):  *“tuberous sclerosis complex”, “bourneville”, “rett”* |
| Pharmaceutical Benefits Scheme (PBS) | <http://www.pbs.gov.au/pbs/home> | 26^th^ January 2021 | The webpage was searched for the following terms (using the general search function):  *"tuberous sclerosis complex”, “bourneville”, “rett”* |
| Canadian Agency for Drugs and Technologies in Health (CADTH) | [https://cadth.ca](https://cadth.ca/) | 29^th^ January 2021 | The webpage was searched for the following terms (using the general search function):  *"tuberous sclerosis complex”, “bourneville”, “rett”* |
| Ministerio de Sanidad, Consumo y Bienestar Social (MSCBS) | <http://www.mscbs.gob.es/home.htm> | 1^st^ February 2021 | The webpage was searched for the following terms (using the general search function):  *“tuberous sclerosis complex”, “esclerosis tuberosa”, “bourneville”, “rett”* |
| Agenzia Italiana del Farmaco (AIFA) | <http://www.agenziafarmaco.gov.it> | 1^st^ February 2021 | The webpage was searched for the following terms (using the general search function):  *"Sclerosi tuberosa”, “Sindrome di Bourneville”, “Sindrome de Rett”* |
| Haute Autorité de Santé (HAS) | [https://www.has-sante.fr/portail](https://www.has-sante.fr/portail/) | 29^th^ January 2021 | The webpage was searched for the following terms (using the general search function):  *"La sclérose tubéreuse de Bourneville”, “STB”* |
| Gemeinsamer Bundesausschuss (G-BA) | [https://www.g-ba.de](https://www.g-ba.de/) | 29^th^ January 2021 | The webpage was searched for the following terms (using the general search function):  *"tuberous sclerosis complex”, “Tuberöse Sklerose”, “Knollensklerose”, “Bourneville”, “rett”* |
| Bundesamt für Gesundheit (BAG) | <https://www.bag.admin.ch/bag/de/home.html> | 29^th^ January 2021 | The webpage was searched for the following terms (using the general search function):  *"tuberous sclerosis complex”, “Tuberöse Sklerose”, “Knollensklerose”, “Bourneville”, “rett”* |
| State of Israel – Ministry of Health | <https://www.health.gov.il/English/Pages/HomePage.aspx> | 2^nd^ February 2021 | The webpage was searched for the following terms (using the general search function):  *”tuberous sclerosis complex”, “bourneville”, “rett”*  The webpage was also searched in Hebrew using the following terms:  תסמונת רט  טוברוס סקלרוזיס  קו מנחה  הדרכה  ישראל |
| Ministry of Health, Labour and Welfare (MHLW) | [https://www.mhlw.go.jp/english](https://www.mhlw.go.jp/english/) | 3^rd^ February 2021 | The webpage was searched for the following terms (using the general search function):  *“tuberous sclerosis complex”, “bourneville”, “rett”*  The webpage was also searched in Japanese using the following terms:  (レット症候群), (Rett症候群), (ガイドライン, ガイド), (指導), (指針), (アドバイス) |
| Institute for Clinical and Economic Review (ICER) | <https://icer-review.org/> | 25^th^ January 2021 | The webpage was searched for the following terms (using the general search function):  *“tuberous sclerosis complex”, “bourneville”, “rett”* |

^a^All results were screened for relevance, with the exception of Google searches, where the first three pages of search results for each search were screened for relevance. AAN, American Academy of Neurology; AES, American Epilepsy Society; AIFA, Agenzia Italiana del Farmico; BAG, Bundesamt für Gesundheit; CADTH, Canadian Agency for Drugs and Technologies in Health; G-BA, Gemeinsamer Bundesausschuss; HAS, Haute Autorité de Santé; ICER, Institute for Clinical and Economic Review; ILAE, International League Against Epilepsy; MHLW, Ministry of Health, Labour and Welfare; MSCBS, Ministerio de Sanidad, Consumo y Bienestar Social; NICE, National Institute for Health and Care Excellence; NORD, National Organisation for Rare Disorders; PBS, Pharmaceutical Benefits Scheme; SMEI, severe myoclonic epilepsy of infancy.

Table S2. List of included guidelines

| Title | Developing bodies | Region | Date (latest revision) | Indication(s) | Reference |
| --- | --- | --- | --- | --- | --- |
| Epilepsies: the diagnosis and management of epilepsies in adults and children in primary and secondary care | NICE | UK | January 2021 | Tuberous sclerosis complex | NICE, 2021([1](#_ENREF_1)) |
| Provincial Guidelines for the Management of Medically-Refractory Epilepsy in Adults and Children Who Are Not Candidates for Epilepsy Surgery | EITF, Critical Care Services Ontario and Provincial Neurosurgery Ontario | Canada (Ontario) | March 2016 | Tuberous sclerosis complex | EITF, 2016([2](#_ENREF_2)) |
| Tuberous Sclerosis Complex Surveillance and Management: Recommendations of the 2012 International Tuberous Sclerosis Complex Consensus Conference | NR | International | October 2013 | Tuberous sclerosis complex | Krueger DA., Northrup H. et al. 2013([3](#_ENREF_3)) |
| Epilepsies in Children and Young People: Investigative Procedures and Management | SIGN | UK (Scotland) | May 2021 | Tuberous sclerosis complex | SIGN, 2018([4](#_ENREF_4)) |
| Guideline for Management of Children with Epileptic Seizures in British Columbia | Division of Neurology, British Columbia Children's Hospital (in collaboration with the Departments of Paediatrics and Psychology and the British Columbia Paediatric Society) | Canada (British Columbia) | April 2011 | Tuberous sclerosis complex | Farrell K., Connolly M. et al. 2011([5](#_ENREF_5)) |
| ILAE Lecture Notes: From Channels to Commissioning - A Practical Guide to Epilepsy; Chapter 30 - Drug Treatment of Paediatric Epilepsy | ILAE (UK Chapter) and Epilepsy Society; sponsored by UCB Pharma and Eisai | UK | 2017 (15th edition; originally published 1987) | Tuberous sclerosis complex | Appleton R.E. and Cross H., 2017([6](#_ENREF_6)) |
| The UK Guidelines for the Management and Surveillance of Tuberous Sclerosis Complex | TSA | UK | March 2019 | Tuberous sclerosis complex | Amin S. et al. 2019([7](#_ENREF_7)) |
| Management of Epilepsy Associated with Tuberous Sclerosis Complex: Updated Clinical Recommendations | 2018 European Paediatric Neurology Society | International (Europe) | May 2018 | Tuberous sclerosis complex | Curatolo P. et al. 2018([8](#_ENREF_8)) |
| National Diagnostic and Treatment Protocol: Rett's Disease and Related Syndromes | HAS | France | April 2017 | Rett syndrome | Bahi-Buisson N. et al. 2017([9](#_ENREF_9)) |
| Tuberous Sclerosis: Clinical Spectrum, Diagnosis, Treatment | NR | France | February 2008 | Tuberous sclerosis complex | Chiron C. 2008([10](#_ENREF_10)) |
| Treatment of Epilepsy in Children | Società Italiana di Neuropsichiatria dell'Infazia e dell'Adolescenza (SINPIA) and University Hospital (AOU) Meyer | Italy | January 2017 | Tuberous sclerosis complex | Guerrini R. et al. 2017([11](#_ENREF_11)) |
| Diagnosis and Treatment of Epilepsies: Guideline for the Tuscany Region (SNLG) | Tuscany Regional Health Council | Italy (Tuscany) | 2014 (Revised version of the 2006 original) | Tuberous sclerosis complex | Campostrini, R. et al. 2014([12](#_ENREF_12)) |
| Updated Practical Manual for the Diagnosis and Treatment of Epilepsy | NR | Italy | July 2012 | Tuberous sclerosis complex | Anzellotti F. and Onofrj M., 2012([13](#_ENREF_13)) |
| History of Rett Syndrome | Ministry of Health Labour and Welfare, Rett Syndrome Research Group | Japan | 2015 | Rett syndrome | Nabatame S. and Ito M., 2015([14](#_ENREF_14)) |
| Tuberous Sclerosis Complex Diagnosis Criteria and Treatment Guideline: Revised Version | Japan Tuberous Sclerosis Complex Society and Refractory Disease Policy Study Group formed by Ministry of Health, Labour and Welfare and Japanese Dermatological Association | Japan | 2018 | Tuberous sclerosis complex | Kaneda M. et al. 2018([15](#_ENREF_15)) |
| Childhood Epilepsy Medical Treatment Guide Understood by Flow Chart | Okayama University | Japan | 2011 | Tuberous sclerosis complex | Ohtsuka Y. et al. 2011([16](#_ENREF_16)) |
| Andalusian Epilepsy Guide 2015: Diagnosis and Treatment of Epilepsy in Children and Adults | Andalusian Epilepsy Society | Spain (Andalusia) | 2015 | Tuberous sclerosis complex | Sánchez-Álvarez J.C. et al. 2015([17](#_ENREF_17)) |
| Tuberous Sclerosis Complex: Cancer in Palimalformative Genetic Syndromes Working Group | Hospital Vall d'Hebron Barcelona | Spain | July 2015 | Tuberous sclerosis complex | Boronat. S et al. 2015([18](#_ENREF_18)) |
| Syndromes and Support. An Overview from Science and from Associations. | Spanish Confederation of Organisations in favour of People with Intellectual Disabilities - FEAPS; OBRA Social. | Spain | 2006 | Rett syndrome  Tuberous sclerosis complex | Piedecasas P.S. et al. 2006([19](#_ENREF_19)) |
| Diagnostic and therapeutic recommendations of the SEN 2019 | Sociedad Española de Neurologia | Spain | 2019 | Tuberous sclerosis complex | López González F.J. et al. 2019([20](#_ENREF_20)) |
| Evidence-based guideline update: Medical treatment of infantile spasms | AAN, Child Neurology Society | US | June 2012 | Tuberous sclerosis complex | Go C.Y. et al. 2012([21](#_ENREF_21)) |
| Treatment of pediatric epilepsy: European expert opinion, 2007 | NR | International  (Europe) | December 2007 | Tuberous sclerosis complex | Wheless J.W. et al. 2007([22](#_ENREF_22)) |
| Summary of recommendations for the management of infantile seizures: Task Force Report for the ILAE Commission of Pediatrics | ILAE, Commission for Pediatrics | International | June 2015 | Tuberous sclerosis complex | Wilmshurst J.M. et al. 2015([23](#_ENREF_23)) |
| Treatment of Pediatric Epilepsy: Expert Opinion, 2005 | NR | US | November 2005 | Tuberous sclerosis complex | Wheless J.W. et al. 2005([24](#_ENREF_24)) |

AAN: American Academy of Neurology; BPNA, British Paediatric Neurology Association; DGN, Deutsche Gesellschaft für Neurologie; EITF, Epilepsy Implementation Task Force; HAS, Haute Autorité de Santé; ILAE, International League Against Epilepsy; MSCBS, Ministerio de Sanidad, Consumo y Bienestar Social; NHS, National Health Service; NICE, National Institute for Health and Care Excellence; NR, not reported; SIGN, Scottish Intercollegiate Guidelines Network; SINPIA, Società Italiana di Neuropsichiatria dell'Infazia e dell'Adolescenza; TGA, Therapeutic Goods Administration; TSA: Tuberous Sclerosis Association; UK, United Kingdom; US, United States.

Table S3. Treatment line-specific recommendations for TSC

| **Treatment line-specific recommendations (positive)** | | |  |
| --- | --- | --- | --- |
| **First-line** | **23** | **Second-line** | **19** |
| Vigabatrin | 15 | ACTH | 6 |
| Prednisolone | 2 | Topiramate | 4 |
| Tetracosactide | 2 | Prednisolone | 2 |
| ACTH | 2 | Sodium valproate | 2 |
| Carbamazepine | 1 | Carbamazepine | 1 |
| Topiramate | 1 | Corticosteroids | 1 |
|  |  | Oxcarbazepine | 1 |
|  |  | Prednisone | 1 |
|  |  | Zonisamide | 1 |
|  |  |  |  |
| **Treatment line-specific recommendations (negative)** | | |  |
| **First-line** | **0** | **Second-line** | **0** |
| N/A |  | N/A |  |

No treatment line-specific recommendations were identified for Rett syndrome. ACTH, adrenocorticotropic hormone, TSC, tuberous sclerosis complex; N/A, not applicable.

**SUPPLEMENTARY REFERENCES**

1. National Institute for Health and Care Excellence. Epilepsies: Diagnosis and Management (CG137) [Internet]. 2018 [cited October 11 2019]. Available from: <https://www.nice.org.uk/guidance/cg137>.

2. Epilepsy Implementation Task Force (EITF). Provincial Guidelines for the Management of Medically-Refractory Epilepsy in Adults and Children Who Are Not Candidates for Epilepsy Surgery. Critical Care Services Ontario [Internet]. 2016 [cited October 13 2019]. Available from: <https://www.criticalcareontario.ca/EN/Epilepsy%20Guideline%20Series/Prov%20Guidelines%20for%20Management%20of%20MRE%20in%20Adults%20Children%20not%20candidates%20for%20Surgery_EN.pdf>.

3. Krueger DA, Northrup H, Northrup H, Krueger DA, Roberds S, Smith K, et al. Tuberous Sclerosis Complex Surveillance and Management: Recommendations of the 2012 International Tuberous Sclerosis Complex Consensus Conference. Pediatric Neurology. 2013;49(4):255-65.

4. Scottish Intercollegiate Guidelines Network. Epilepsies in Children and Young People: Investigative Procedures and Management. Draft Version [Internet]. 2018 [cited July 2018; draft version no longer publically available]. Available from: <www.sign.ac.uk>.

5. Farrell K, Connolly M. Guideline for Management of Children with Epileptic Seizures in British Columbia. Child Health British Columbia [Internet]. 2011 [cited October 2019]. Available from: <https://www.childhealthbc.ca/sites/default/files/Management%20of%20Children%20with%20Epileptic%20Seizures%202011.pdf>.

6. Appleton RE, Cross HJ. ILAE Lecture Notes: From Channels to Commissioning - A Practical Guide to Epilepsy; Chapter 30 - Drug Treatment of Paediatric Epilepsy. ILAE and Epilepsy Society [Internet]. 2017 [cited October 2019]. Available from: <https://www.epilepsysociety.org.uk/sites/default/files/attachments/Chapter30AppletonCross2015.pdf>.

7. Amin S, Kingswood JC, Bolton PF, Elmslie F, Gale DP, Harland C, et al. The UK guidelines for management and surveillance of Tuberous Sclerosis Complex. QJM: An International Journal of Medicine. 2018;112(3):171-82.

8. Curatolo P, Nabbout R, Lagae L, Aronica E, Ferreira JC, Feucht M, et al. Management of epilepsy associated with tuberous sclerosis complex: Updated clinical recommendations. European journal of paediatric neurology : EJPN : official journal of the European Paediatric Neurology Society. 2018;22(5):738-48.

9. Bahi-Buisson N, Philippe C, and members of the working group. Protocole national de diagnostic et de soins (PNDS): Syndromes De Rett Et Apparentés. 2017 [cited October 2019]. Available from: <https://www.has-sante.fr/upload/docs/application/pdf/2017-05/dir4/pnds_-_syndromes_de_rett_et_apparentes_2017-05-03_14-15-56_224.pdf>.

10. Chiron C. Sclérose tubéreuse de Bourneville : spectres cliniques, démarche diagnostique, prise en charge pratique. La Lettre du Neurologue. 2008;XII(1-2):16-20.

11. Guerrini R, Chiamenti G, Mugelli A, Ruggieri M, Lubrano R, Provinciali L, et al. Linee Guida: Epilessie pediatriche. Associazione Italiana Contro l'Epilessia [Internet]. 2017 [cited October 2019]. Available from: <http://www.aice-epilessia.it/index.php?option=com_content&view=article&id=176:linee-guida-epilessie-in-eta-pediatrica&catid=1:banner>.

12. Campostrini R, Amantini A, Balestri P, Barba C, Bianchi A, Chicchetti B, et al. SNLG – Diagnosi e trattamento delle epilessie. Regione Toscana [Internet]. 2014 [cited October 2019]. Available from: <http://www.regione.toscana.it/documents/10180/320308/Diagnosi+e+trattamento+delle+epilessie+linea+guida+aggiornamento+2014/82e824af-af27-4f34-83ec-13e0bb67c78b>.

13. Anzellotti F, Onofrj M. Nuovo manuale pratico per la diagnosi e la cura delle epilessie [Internet]. 2012 [cited October 2019]. Available from: <https://issuu.com/gruppo.opera/docs/epilessia>.

14. Nabatame S, Ito M. レット症候群診療ガイドブック (History of Rett Syndrome). Osaka University Press 2015.

15. Kaneda M, Mizuguchi M, Hatano T, Seyama K, Hino M, Nishigori C. 結節性硬化症の診断基準及び治療ガイドライン―改訂版― (Diagnostic Criteria and Treatment Guideline for Tuberous Sclerosis Complex -Revised Edition-). JSHI Journal. 2018;128(1):1-16.

16. Ohtsuka Y, Kobayashi K, Yoshinaga H. フローチャートでわかる小児てんかん診療ガイド (Childhood Epilepsy Medical Treatment Guide Understood by Flow Chart). Shindan to Chiryo Sha 2011.

17. Sánchez-Álvarez J, Ruiz-Giménez J, Roldán Aparicio S, Serrano-Castro P, Arenas Cabrera C, Camino León R, et al. Guía Andaluza de la Epilepsia 2015: Diagnóstico y tratamiento de la epilepsia en niños y adultos [Internet]. 2015 [cited October 2019]. Available from: <https://escueladepacientes.es/images/Pdfs/SADE%20-%20Gu%C3%ADa%20Andaluza%20de%20Epilepsia%202015.pdf>.

18. Boronat S, Sábado C, Vendrell T, Martínez-Glez V. Complejo esclerosis tuberosa [Internet]. 2015 [cited October 2019]. Available from: <https://www.ciberer.es/media/445781/gt-csgp-cet.pdf>.

19. Piedecasas PS, García Fuentes M, Antonio del Barrio J, Palomera Martín R, Sangrador Martínez B, Saínz Hernández C, et al. Síndromes y apoyos. Panorámica desde la ciencia y desde las asociaciones. FEAPS [Internet]. 2006 [cited October 2019]. Available from: <http://riberdis.cedd.net/bitstream/handle/11181/3373/S%C3%ADndromes%20y%20apoyos.pdf?sequence=1>.

20. Neurología SEd. Recommendaciones diagnóstico-terapeuticas da la SEN2019. 2019 [May 2021]]. Available from: <http://epilepsia.sen.es/wp-content/uploads/2020/06/Recomendaciones-Epilepsia-SEN-2019.pdf>.

21. Go CY, Mackay MT, Weiss SK, Stephens D, Adams-Webber T, Ashwal S, et al. Evidence-based guideline update: medical treatment of infantile spasms. Report of the Guideline Development Subcommittee of the American Academy of Neurology and the Practice Committee of the Child Neurology Society. Neurology. 2012;78(24):1974-80.

22. Wheless JW, Clarke DF, Arzimanoglou A, Carpenter D. Treatment of pediatric epilepsy: European expert opinion, 2007. Epileptic Disord. 2007;9(4):353-412.

23. Wilmshurst JM, Gaillard WD, Vinayan KP, Tsuchida TN, Plouin P, Van Bogaert P, et al. Summary of recommendations for the management of infantile seizures: Task Force Report for the ILAE Commission of Pediatrics. Epilepsia. 2015;56(8):1185-97.

24. Wheless JW, Clarke DF, Carpenter D. Treatment of pediatric epilepsy: expert opinion, 2005. Journal of child neurology. 2005;20 Suppl 1:S1-56; quiz S9-60.
